# Practice and Evaluation of Competence in Assessment of Arterial Circulation of the Lower Limbs among Medical Students and Physicians in Training – A Systematic Review

**DOI:** 10.1177/23821205241303560

**Published:** 2024-12-05

**Authors:** Martin Macek, Frida Eek, Axel Wrede, Talha Butt, Stefan Acosta

**Affiliations:** 1 Vascular Diseases, Department of Clinical Sciences, 174435 Lund University, Malmö, Sweden; 2 Department of Health Sciences, 174425 Lund University, Lund, Sweden; 3 Vascular Centre, Skåne University Hospital, Malmö, Sweden

**Keywords:** Vascular examination, clinical practice, medical education, lower extremity arterial disease, lower extremity circulation

## Abstract

**Introduction:**

A recent study on patients with acute lower limb ischemia showed that the proportion of inadequate examination of lower extremity circulation was associated with higher rate of amputation and death. The aim of this systematic review was to explore evidence for how practical competence in performing a peripheral vascular status of the lower limb among medical students and junior doctors should be taught and examined.

**Methods:**

The systematic review followed PRISMA guidelines and was published in PROSPERO. Articles were searched for in PubMed, Cochrane Library and Embase. The result was processed by two researchers. After title- and abstract screenings, articles were scrutinized in full text for inclusion, result extraction, risk of bias assessment through Medical Education Research Study Quality Instrument (MERSQI), and evidence grading with the GRADE approach.

**Results:**

Thirteen studies were included. Two studies were randomized controlled trials (RCTs). Study samples varied between medical students (n = 9), junior doctors (n = 3) and residents (n = 3). Interventions varied between theoretical, practical, repetitive training, feedback-based learning, and clinical experience. Assessed measurements (outcomes) were ankle-brachial index (ABI) (n = 9), theoretical knowledge (n = 4), pulse palpation (n = 1) and complete vascular status (n = 1). Experienced residents had better theoretical knowledge than inexperienced residents, but performance of the entire ABI procedure without any mistake according to guidelines was inadequate in both groups. One RCT showed that experimental training significantly increased ability to perform ABI measurements, but this ability decreased after six months without repetition.

**Conclusion:**

Theoretical training alone is not sufficient in ensuring proficiency in vascular examination of the lower limbs. Continuous practice and clinical exposure are crucial to maintain proficiency in performing vascular examination of the lower limbs. Data is limited and heterogenous. The level of certainty for the evidence was judged to be very low.

## Introduction

Lower extremity artery disease (LEAD) is caused by chronic progressive atherosclerosis of the lower limbs resulting in decreased arterial circulation in the affected limb. Risk factors for LEAD are age, diabetes, hypertension, hypercholesterolemia, high body mass index (BMI) and smoking.^1^ Most LEAD cases are asymptomatic and can be diagnosed by measuring the patient's ankle brachial index (ABI).^2^ Acute limb ischemia (ALI) occurs when circulation to the limb is acutely disrupted and where symptoms have not yet persisted for more than two weeks. The symptoms for ALI differ from chronic limb-threatening ischemia (CLTI), and are clinically generally characterized by the “six Ps”; *Pain, Pallor, Pulselessness, Poikilothermia, Paraesthesia and Paralysis*.^3^ ALI is a medical emergency and requires prompt diagnosis and treatment to prevent loss of limb or death.^4^

Clinical history and clinical examination are important diagnostic tools regarding LEAD. In addition to this, measurement of the patient's ankle pressure and calculation of their ABI is an inexpensive and minimally invasive bedside examination that is valid for cardiovascular (CV) risk assessment. ABI examination requires good training and is conducted using a blood pressure cuff with its manometer positioned above the ankle and hand-held Doppler ultrasound probe placed above either of the two main foot arteries, amplifying sound of arterial blood flow.^5^ Toe brachial index measurement using a smaller blood pressure cuff around the great toe and a laser Doppler flow detector distal to the cuff, can be used when patients have noncompressible arteries in the lower leg, which is more present in those with diabetes mellitus^4^ and chronic kidney disease.^6^ Duplex ultrasound, digital subtraction angiography (DSA), computed tomography angiography (CTA) and magnetic resonance angiography (MRA) can further be used to visualize and more accurately examine the affected arteries.^5^ Exposure to vascular surgery training in medical schools vary considerably.^7^ One in four final year medical students in the United Kingdom had never conducted a vascular clinical examination and 26% experienced low confidence in performing the ABI examination.^8^

A recent population-based study showed that a satisfactory first clinical examination of the arterial circulation in the lower extremities and ABI’s in patients with ALI at admission at Skåne University Hospital was unsatisfactorily low. Better clinical examinations were associated with favorable outcome at 1 year.^9^

The aim of this systematic review was to explore and summarize the existing evidence for how practical competence of performing a peripheral vascular status of the lower limb among medical students and junior doctors is best taught and examined.

## Methods

The systematic review was presented in accordance with PRISMA (Preferred Reporting Items for Systematic Review and Meta-Analysis) guidelines^10^ and the PRISMA 2020 Checklist (Appendix, Supplementary Table 1) and PRISMA 2020 Abstract checklist (Appendix, Supplementary Table 2) to standardize reporting. Predetermined target populations, inclusion and exclusion criteria and outcome measures, were published as a protocol in the International Prospective Register of Systematic Reviews (PROSPERO) on first of May 2022 with registration number CRD42022322338.^11^

### Eligibility criteria

The study samples were determined to consist of medical students, junior doctors, medical interns, surgical interns, medical residents or surgical residents. The interventions/exposures were training and/or examination of practical skills in vascular leg status, during medical school, internship years or residency training. All sorts of practical training were included. The comparisons conducted were between exposed and non-exposed to the intervention or within the same sample before and after exposure to the intervention. Consultants, specialist physicians or vascular technicians could be the reference in comparative studies. The outcome was quality/practical competency in examination of arterial circulation in the lower limbs according to the judgements of the individual studies, both in simulated and clinical settings. The measures of effect were defined by the individual studies. There were no restrictions on study design of the articles in the search strategy.

### Search strategy

The search strategy was formed together with a research librarian at the Medical Faculty, Lund University, to fit the eligibility criteria. Articles were searched for in PubMed, Cochrane Library, and Embase on May 22^nd^, 2023 (Appendix, Supplementary Table 3). The search strategy was formed with search templates for the individual search engines to ensure the greatest accuracy possible. There were no language restrictions in the literature search.

### Study selection

Search hits were imported and managed in the online tool Covidence^®^. Duplicates were automatically removed. Two reviewers (MM, AW), independently reviewed article titles and abstracts and the non-relevant articles were excluded. The remaining articles were scrutinized in full text by the same reviewers for final inclusion or exclusion from the review. Disagreements in selection of articles were resolved in a consensus-based approach together with a third reviewer (SA). Reference lists of included studies were screened for potential additional articles. Review articles in the study selection were not included, but the articles that comprised their result were sought for, and if relevant to the inclusion criteria, were included in this study.

### Data collection

Information about each study’s sample, intervention, control and outcome and summary of results was extracted and presented in tables.

### Bias assessment

Using the Medical Education Research Study Quality Instrument (MERSQI),^12^ three reviewers (MM, SA, FE) independently assessed study quality and risk of bias of the included articles. MERSQI is a validated tool designed for medical education research. The scoring system tool consists of eight domains: study design (0-3 points), sampling (0–1.5 points), sampling response rate (0-1.5 points), type of data (0-3 points), validity evidence for evaluation instrument (0-3 points), sophistication of data analysis (0-2 points), appropriateness of data analysis (0-1 points) and outcome (0-3 points). A maximum of 18 points can be given to an article. Scoring of the articles according to MERSQI was performed in a consensus-based approach (MM, SA, FE) with support from extended elaboration and explanation of the domains.^13^

### Evidence grading

Evidence grading was conducted according to the Grading of Recommendations, Assessment, Development and Evaluation (GRADE) approach.^14^ According to this evidence grading instrument, the study design decides each study’s start level of evidence, where RCT starts at the top level of evidence (high) whereas non-RCTs start with an evidence level of low. Five domains can each lower the evidence level one step: risk of bias, inconsistency, indirectness, imprecision or publication bias. Three domains can raise the evidence level; a large effect size, plausible confounding or a dose-response gradient.^15^ Finally, a grade on a four-step scale is given to the summed evidence level of the studies included in the systematic review ranging from very low to high.

### Study reporting

Studies were reported according to *PICO*, a mnemonic for *population*, *interventions*, *control* and *outcome*, to encapsulate specifications of the included studies in the review according to Cochrane handbook.^16^

### Statistical analysis

Meta-analysis was not appropriate due to heterogenous study designs, exposures, and outcomes. Complementary statistical analyses were performed in cases where statistical analyses had not been carried out in studies, and when the result was clear enough for statistical analyses to be performed. This was done for three studies using GraphPad QuickCalcs Web site^17^ for statistical calculations. Within the study by Omarjee et al,^18^ proficient students, having passed all theoretical and practical tests at the final assessment, were re-invited to perform an evaluation at 6 months of follow up to verify acquisition of knowledge and skills of measuring ABI. The number of proficient students in terms of percentage of success at final assessment and six month follow-up was calculated, and within-group comparison was performed with the McNemar's test. The proportion of imprecise ABI measurements (>0.15 mm Hg) by junior doctors with no training and junior doctors with training and feedback, were compared to an experienced vascular technician in the study by Ray et al^19^ Krueger et al^20^ reported that medical students and interns having cared for patients with LEAD self-estimated to have better ability to adequately diagnose LEAD compared to those who only received lectures. Group comparison in these two studies^19,20^ was conducted using the Chi-square test. A two-tailed P value of <0.05 was considered statistically significant.

## Results

### Study selection

**Figure 1.**
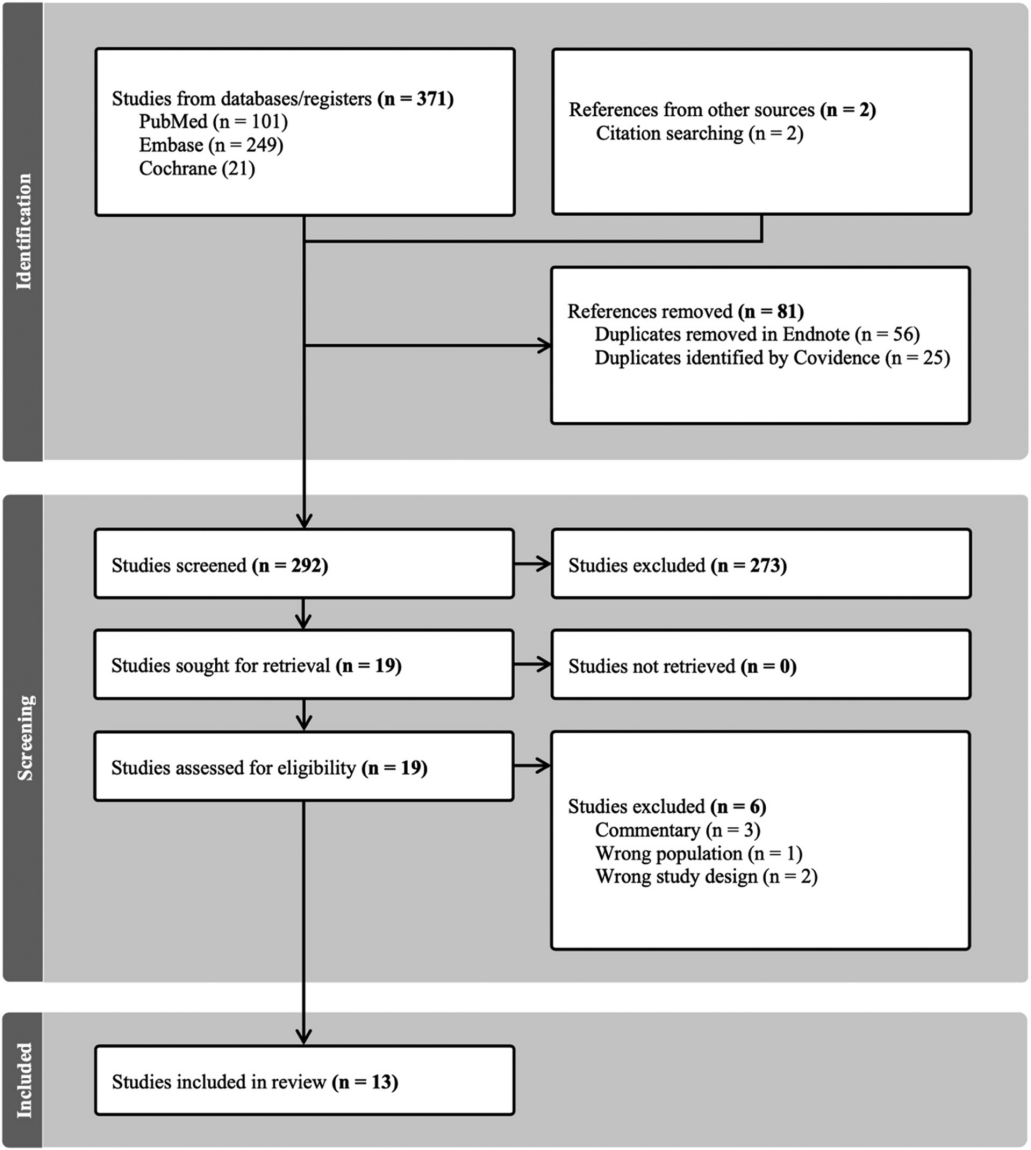
PRISMA table of literature review.

Three hundred and seventy one studies were extracted for processing in Covidence. Twenty-five duplicate studies were identified and automatically removed by Covidence. Through initial title and abstract screening, 273 studies were excluded for not meeting the pre-defined inclusion criteria. Nineteen studies were retrieved in full text for thorough scrutiny and eligibility assessment resulting in further six studies being excluded for not meeting inclusion criteria. A total of 13 studies^18–30^ were included (Figure 1).

### Study characteristics

Two of the 13 studies were randomized controlled trials (RCT), seven were cohort studies (studies with control group n = 3, studies testing same study sample pre and post intervention n = 4) and four were cross-sectional studies. One cohort study was published in the form of a letter to the editor and one cross-sectional study was published in the form of a research letter. The studies were published between the years 1993 and 2021. Two studies were published before year 2000 while four were published during the last five years. The study samples ranged from 4–150 study participants (Table 1).

**Table 1.** Study designs of included studies according to PICO^A^.

ARTICLE	STUDY DESIGN	PROCEDURE	TOTAL N	POPULATION	INTERVENTION	COMPARISON	OUTCOME	MAIN RESULTS
Ahn et al ^28^	Randomized controlled trial.	Pulse palpation of the femoral artery and vein.	150	Medical students. second year.	Vascular ultrasound instruction for localization of vessel.	Group with (n = 71) versus without (n = 79) intervention.	Accuracy in localizing femoral artery by manual palpation.	Students in the intervention group located the femoral pulse more accurately (mean distance from artery 0.46 ± 0.71 cm) than students without intervention (mean distance from artery 1.26 ± 2.06 cm) (p = 0.02*). Students in the intervention group estimated the location of the femoral vein more accurately (mean 89% ± 31) than students without intervention (mean 79% ± 41) (p = 0.093).
Donnou et al ^29^	Randomized controlled trial.	ABI measurement.	30	Medical students. fourth – sixth year.	Practical training on healthy volunteer, patient or didactic learning alone.	Group with (n = 20) versus without (n = 10) intervention.	Proficiency in performing ABI examination and calculation.	At final evaluation, the number of proficient students in didactic learning group had not significantly improved (0/10 proficient at initial evaluation vs 1/10 proficient at final evaluation; p = nonsignificant). After three ABI examinations, the number of proficient students significantly increased (0/20 proficient at initial evaluation vs 11/20 proficient at final evaluation (p < 0.05*). At final evaluation, the number of proficient students significantly differed between didactic learning group and didactic + training group (1/10 in didactic learning alone vs 11/20 in didactic + experimental training (p < 0.05*). At final evaluation, there was no significant difference between students training on healthy individuals versus students training on patients.
Omarjee et al ^18^	Cohort study.	ABI measurement.	11	Medical students. fourth – sixth year who were deemed proficient at ABI measurement and Wyatt score in study 3 (Donnou et al)	Fidelity assessment after 6 months for proficient students in study by Donnou et al ^29^	Group with (n = 10) versus without (n = 1) intervention.	Proficiency in performing ABI examination and calculation.	Among 11 proficient medical students, 4 students passed repeat testing at 6 months (p = 0.0013^B^*)
Congnard et al ^21^	Cohort study.	ABI measurement.	27	Medical students. second year.	Practical training. 1 h lesson.	1 group (n = 27), pre and post intervention.	Accuracy of ABI measurement.	The error of ABI measurements (p = 0.002*) and mean deflation rate (p = 0.001*) were significantly reduced after intervention.
Georgakarakos et al ^26^	Cohort study.	ABI measurement (accuracy and amount of time to perform).	5	Medical students. fourth year.	Repetitive practical training on patients.	1 group (n = 5), pre and post intervention.	Accuracy of ABI measurement and time to perform ABI measurement.	Error of measurement was reduced after repetitive training (20 measurements) in comparison to trainer (Initial difference in ABI 0.32 ± 0.23 vs 0.23 ± 0.07 (p = 0.0002*) and at later stage 0.38 ± 0.05 versus 0.32 ± 0.08, (p = 0.09). Reduction of standard deviation in ABI result of students after repetitive training (initially 0.23 to 0.05 at later stage). Reduction of time needed to complete ABI examination after repetitive training (initially 20 min to 10 min).
Ray et al ^19^	Cohort study	ABI measurement.	4	Junior doctors.	Training session and personalized feedback by experienced ABI technician.	Group with (n = 2) versus without (n = 2). intervention.	Accuracy of ABI measurement.	29% of the ABI measurements obtained by junior doctors without intervention differed more than 0.15 mm Hg from those obtained by vascular technician, compared to 15% of the ABI measurements after intervention (p = 0.08^B^). The differences in ABI between trained doctors and the vascular technician were distributed more normally. The mean difference in ABI between junior doctors with intervention and vascular technician was significantly less at the dorsalis pedis compared to junior doctors without intervention (p = 0.042^B*^).
Wyatt et al ^27^	Cohort study.	ABI performance, calculation and theoretical knowledge.	29	Internal medicine residents. first – third year.	Personalized feedback on errors after initial ABI measurement and common errors of the group.	1 group (n = 29) pre and post intervention.	ABI measurement procedure and knowledge on ABI calculation and interpretation.	4% of the residents completed ABI measurement without an error (mean score 4.55 ± 2.95, range 1–15) at baseline compared to 50% of the residents (mean score 13.88 ± 1.60, range 1–15) following the educational intervention (p < 0.0001*).
Kuo et al ^22^	Cohort study. Letter to the editor.	Theoretical knowledge about vascular surgery-	7	Medical students. third year.	Week long course including lectures, reading instructions, reading publications and virtual skills laboratory of suturing techniques with real time feedback.	1 group (n = 7) pre and post intervention.	Questionnaire covering vascular surgery topics including vascular physical examination.	Students had improved performance in terms of improved mean score post intervention (16.6 ± 3.4) compared to pre intervention (11.6 ± 3.5) (p < 0.03*).
Monti et al ^30^	Cohort study.	ABI.	12	Medical students.	Experience in terms of number of ABI exams performed.	Experienced (n = 6) versus all medical students (n = 12).	Accuracy of ABI measurement.	Experienced medical students were not significantly better than the whole group (experienced and non-experienced). There was no significant correlation between number of exams performed and mean ABI difference between student and angiologist.
Lanéelle et al ^23^	Cross-sectional study. Research letter.	Theoretical knowledge on ABI, PEABI, TBI.	44	Vascular medicine residents. first year.	Experience of performing ABI, post -exercise ABI (PEABI) and toe brachial index (TBI) procedures. A resident performed the examination	Group with versus without intervention.	Knowledge about ABI, PEABI and TBI procedures.	Experienced residents (having performed the separate procedure at least once before) were statistically more competent (score of 50% or more on questionnaire) compared to inexperienced residents in all three tests; ABI (79% vs 50%), PEABI (67% vs 42%) and TBI (53% vs 31%) (p < 0.001*).
Chaudru et al ^24^	Cross-sectional study.	ABI.	68	Medicine and cardiology residents.	Experience in terms of having performed more than 20 ABI measurements in clinical practice.	Group with (n = 26) versus without (n = 42) intervention.	Knowledge on the ABI procedure.	Experienced residents had a significantly (p < 0.05*) higher total score compared with inexperienced residents.
Endean et al ^25^	Cross-sectional study.	Vascular examination and theoretical knowledge.	50	Medical students, junior doctors and interns.	Experience in terms of level of education (third year medical students, novice interns and interns after 1 year of surgical residency).	Between the three groups (medical students n = 20, junior doctors n = 23, interns n = 7) as well as within groups if vascular rotation/preceptor versus not.	Overall performance of physical vascular status and knowledge on ABI procedure.	Surgical interns after 1 year of surgical residency (mean score 62 ± 4) performed better than third year medical students (mean score 43 ± 3) and novice interns (mean score 39 ± 3) (p = 0.0005*). Medical students with a vascular surgery preceptor during their surgical clerkship compared to peers with non-surgical preceptor had significantly better knowledge about the ABI procedure (62% ± 7 vs 32% ± 4), p = 0.0011. These students also had a significantly better overall score (61 ± 3 vs 38 ± 3), (p = 0.0006*).
Krueger et al ^20^	Cross-sectional study.	Self-reported understanding and ability to diagnose LEAD.	55	Medical students or interns.	Having cared for patient with LEAD.	Group with (n = 20) versus without (n = 35) intervention.	Self-reported level of understanding of ability to diagnose LEAD.	Medical students and interns having cared for patients with LEAD in clinical setting and received lecture on LEAD self-estimated to have a better ability to adequately diagnose LEAD (44.4%) compared to students having solely received lectures on LEAD (28.6%) (p = 0.32^B^).

A – Population, intervention, control, outcome.

B – P-value from complementary statistical analyses.

LEAD – Lower Extremity Arterial Disease

* – significant result.

The most assessed aspect of the vascular examination (outcomes) was ankle-brachial pressure index (ABI) (n = 9). General knowledge on vascular examination (n = 2), knowledge about vascular surgery (including diagnostics) (n = 1) and pulse palpation of the femoral arteries (n = 1) were also measured. The PICO of the included studies in the review are outlined in Table 1.

### Bias assessment

The MERSQI score (Table 2) of the studies (n = 13) ranged between 5 and 14.5 (mean score 11.4). Two articles were RCTs, awarding them a full score of 3 on the study design domain. Almost all studies were performed in one institution, except for two studies which were conducted as a survey. Data was collected in an objective manner, by an external examiner or by assessing knowledge in a theoretical test, while one study was based on self-reported assessments.

**Table 2.** MERSQI^A^ score.

DOMAIN OPTION (SCORE)	AHN ^28^	DONNOU ^29^	OMARJEE ^18^	CONGNARD ^21^	GEORGAKARAKOS ^26^	RAY ^19^	WYATT ^27^	KUO ^22^	MONTI ^30^	LANéELLE ^23^	CHAUDRU ^24^	ENDEAN ^25^	KRUEGER ^20^
Study Design Singe-group cross sectional or single-group post test only (1) Single-group pre/post-test (1.5) Nonrandomized, two groups (2) Randomized controlled trial (3)	3	3	3	2	1.5	2	1.5	1.5	1	1	1	1	1
Sampling 1 institution (0.5) 2 institutions (1) 3 or more institutions (1.5)	0.5	0.5	0.5	0.5	0.5	0.5	0.5	0.5	0.5	1.5	1.5	0.5	0.5
Sampling response rate <50% or not reported (0.5) – 74% (1) ≥75% (1.5)	1.5	1.5	1.5	0.5	1.5	1.5	1.5	0.5	0.5	1.5	1.5	0.5	0.5
Type of data Assessment by study participant (1) Objective (3)	3	3	3	3	3	3	3	3	3	3	3	3	1
Validity evidence for evaluation instrument (summed score) Content (1) Internal structure (1) Relationship to other variables (1)	1	2	2	0	2	1	1	0	1	0	1	1	0
Data analysis: Sophistication Descriptive analysis only (1) Beyond descriptive analysis (2)	2	2	1	2	2	2	2	2	2	2	2	2	1
Data analysis: Appropriate Data analysis appropriate for study design and type of data (1)	1	1	1	1	0	1	1	0	1	1	1	1	0
Outcome Satisfaction, attitudes, perceptions, opinions, general facts (1) Knowledge, skills (1.5) Behaviors (2) Patient/health care outcome (3)	1.5	1.5	1.5	1.5	1.5	1.5	1.5	1.5	1.5	1.5	1.5	1.5	1
Total MERSQI Score	13.5	14.5	13.5	10.5	12	12.5	12	9	10.5	11.5	12.5	10.5	5
Blinding of assessment	Yes, single blinded.	Yes, single blinded.	Yes, single blinded.	Yes, single blinded.	Yes, single blinded.	Yes, single blinded.	Yes, single blinded.	N/A	Yes, double blinded.	N/A	N/A	Yes, single blinded.	N/A

A Medical Education Research Study Quality Instrument

N/A – not applicable. This information was not possible to obtain from the article.

### Quality of evidence

All studies but two^20,30^ supported a positive effect of theoretical knowledge and practical training on competencies of examination of the circulation in the lower extremity indicating some degree of inconsistency. The populations, interventions and outcomes differed between the studies, restricting indirect comparisons, yielding a very low degree of indirectness. Most studies had few participants, resulting in a large imprecision of results. There were two randomized studies and one prospective study based on one of the RCTs. These three studies had a score of 14.5,^29^ and 13.5^18,28^ points using the risk of bias assessment in the MERSQI tool (Table 2), suggesting lower risk of bias. The remaining ten studies had MERSQI scores ranging from 5 to 12.5, reflecting higher risk of bias. No meta-analysis or pooling of data from the 13 individual studies was possible. Altogether, the level of certainty for the summarized evidence according to the GRADE approach was downgraded to very low.

### Study findings

#### Practical skills in performing ABI examination

Among seven studies reporting on practical ability to perform an accurate ABI procedure,^18,19,21,26,27,29,30^ two (one of which was an RCT) found that practical training significantly improved the study sample's accuracy in performing the ABI procedure.^29^ In a six month follow-up study to the RCT,^21,29^ proficient medical students underwent a fidelity test after not repeating the ABI procedure, showing a significantly inferior ability to perform ABI procedure proficiently (p = 0.0013) compared to immediately after the educational intervention.^18^ Georgakarakos et al^26^ showed that continuous training on ABI measurement led to decreased difference in ABI value measured between trainee and trainer. Two studies showed that residents and junior doctors receiving personalized feedback from an ABI technician after performing ABI measurements, were significantly more able to perform the ABI procedure accurately after intervention.^19,27^ After a nine-day training program on ABI measurement, medical students’, compared to an experienced angiologist, did not achieve an acceptable diagnostic accuracy to detect LEAD in a risk population.^30^

#### Theoretical knowledge of ABI procedure

Three studies measured theoretical knowledge on the ABI procedure among medical students or residents^23–25^: One survey study found that experienced residents (having performed the separate examinations at least once before) were significantly more competent than inexperienced residents at performing ABI, post-exercise ABI (PEABI) and toe brachial index (TBI).^23^ In the second study, residents were considered experienced if they had performed more than 20 ABI examinations prior to taking the survey. Experienced residents possessed significantly better theoretical knowledge on the ABI procedure than inexperienced residents.^24^ In the third study, medical students with a vascular surgery preceptor during their surgical clerkship in medical school had significantly better knowledge on the ABI procedure compared to peers with non-vascular surgery preceptor.^25^

#### Full vascular status and knowledge on ABI procedure

When performing a bedside vascular status and testing theoretical knowledge on ABI, surgical interns after one year of surgical residency performed significantly better than novice interns and third year medical students.^25^

#### Palpation of femoral artery

Ahn et al^28^ showed that medical students who had received vascular ultrasound instruction were significantly more accurate at localizing the femoral artery when examining patients using manual palpation than those students who only received theoretical training.

#### Theoretical knowledge of vascular surgery

In one of the included studies, medical students had significantly improved theoretical general knowledge on vascular surgery (questionnaire including vascular physical examination) after a week-long online course including lectures, reading instructions, publications and virtual skills laboratory of suturing techniques with real time feedback.^22^

#### Ability to diagnose LEAD

Medical students and interns did not significantly (p = 0.32) self-estimate their ability to better diagnose LEAD if they had cared for patients with LEAD in a clinical setting and received lectures on LEAD than those who had only received lectures.^20^

## Discussion

Out of the 13 studies included in the review, two of them were RCTs with higher levels of evidence and lower risks of bias, however the level of certainty for the evidence of the pooled results was very low. Despite heterogenous interventions and outcome measure among the included studies, most of the articles supported a positive outcome regarding physical vascular examination when study participants had trained practically, repetitively and with individual feedback. The most assessed clinical skill was the ABI measurement which is understandable as it is an outcome that can quite easily be measured and compared to a reference standard.

The two RCTs, Donnou et al and Ahn et al, had the highest level of evidence in this educational area in physical vascular examination.^28,29^ In addition, Omarjee et al^18^ followed up on the results of the first RCT conducted by Donnou et al, further strengthening the evidence of this RCT. Donnou showed that practical training was crucial to increase competence in performing ABI examinations.^29^ Omarjee et al found that without practical repetition, medical students’ ability to perform this examination was decreased. Furthermore, Georgakarakos et al showed that continuous practice on performing the ABI procedure enhanced the medical student's accuracy when performing the examination.^26^

Two studies concluded that junior doctors and residents receiving personalized feedback where more accurate when performing the ABI measurement compared to those who did not.^19,27^ This is supported in several previous studies. One study concluded that medical student's receiving summary feedback from an expert after several practice trials on basic surgical techniques were more proficient at fidelity test after 1 month compared to the control group receiving no feedback.^31^ In another study, medical students receiving expert feedback sustained their efficiency in performing surgical knots after one month of no repetition compared to students who only received basic computerized feedback on the number of movements they made.^32^ This suggests that personalized feedback, preferably from an expert, allowing questions from the novice, leads to better performance of a practical task, and most importantly sustains the newly acquired competence. In general, the studies covering the aspect of practical skills in performing an ABI measurement found that practical training with repetition and personalized feedback was crucial to maintain proficiency.

Resident physicians who were experienced in terms of having performed several ABI examinations in clinical practice had better theoretical knowledge of the examination. This further support that repetitive training is crucial, not only for upholding practical skills but also theoretical knowledge on the procedure.

According to Ahn et al,^28^ training with the use of ultrasound to understand the vascular anatomy of the femoral artery improves medical students’ ability to palpate the femoral pulses. Dinh et al^33^ compared medical students’ performance at Objective Structured Clinical Examinations (OSCE) before and after implementation of an ultrasound curriculum. The results showed a significant increase of first-time pass rates of different physical examinations (blood pressure, abdominal examination) for students who had participated in the ultrasound training. This further supports the idea that ultrasound teaching improves medical students’ ability to perform different physical examinations.

Despite two RCTs among the results, the pooled evidence level was graded to be very low. This was mainly due to poor study designs and a high risk of bias among most articles. Since many of the articles had different PICO, a pooled synthesis in the form of a forest plot^34^ and meta-analysis of the result was not suitable. The studies were in many cases not optimally designed. Many times the study sample was very small, with as few as four participants in the study conducted by Ray et al^19^ The small study samples of the included studies impacted the possibility to obtain statistically significant results, reducing generalizability of the results of this review. Several of the studies were imprecise regarding the aim, such as Monti et al^30^ who mainly investigated upon the accuracy of in-patients ABI measurement by medical students and did not focus particularly on the intervention. Two of the studies were research letters^22,23^ with unknown methodology to the reader lowering study quality. Krueger et al^20^ conducted a study where medical students and residents self-estimated their ability to diagnose LEAD meaning a high risk of bias and a very low study quality. Many of the studies lacked a comparison group. Furthermore, many studies with a comparison group lacked appropriate statistical analyses to effectively compare differences. When regarding the potential limitations of the review process, two main aspects became apparent along the way. Although broad inclusion criteria were needed to make sure that the final study selection was as broad and relevant as possible, this created issues where the main objective for some of the included studies did not fully match the aim. The consequence of this issue resulted in some difficulties in extraction of results. Secondly, the use of MERSQI was more difficult than anticipated. Although most domains were easily applicable, some domains such as the *Validity evidence for evaluation instrument* was less objective. Reed et al^12^ studied the association between funding and quality of published medical education research. Two hundred and ten studies were included, with a MERSQI score ranging from 5 to 16 with a mean of 9.95. When comparing this to the MERSQI distribution of this review (range 5-14.5, mean score 11.4), the risk of bias among the studies in this review did not differ notably from the medical education research field. A Modified Medical Education Research Study Quality Instrument (MMERSQI) has been suggested with amendments and changes^35^ and could potentially be an improved instrument for future risk of bias assessments.

Although there were some limitations in the review process as well as in the designs of the included studies, the rigorous methodology in conducting a systematic review ensured that the evidence field in connection to the specific aim was scrutinized in the most thorough manner possible. By using the risk of bias tool and evidence grading system, the reliability and generalizability of the results could be considered. The registration of the review protocol in PROSPERO as well as use of PRISMA flow chart of the study selection ensured a transparent review process. By conducting this review, knowledge gaps were identified and brought to light. This motivates further studies on how vascular examination should be taught and examined to ensure physicians are properly equipped with the practical skills required to properly diagnose LEAD in the future.

There are no current guidelines on how a full physical vascular status should be conducted, taught or examined at medical programs around the world. Hence, more high quality RCTs, prospective longitudinal studies with long follow-up times, or studies based on expert opinions, perhaps using Delphi methodology,^36^ could improve teaching and examination of competence in physical examination of the arterial circulation in the lower extremities at medical student level. Another step forward would be to develop a valid and reliable examination protocol of vascular status of the lower extremities including ankle-brachial index. Such a specific examination protocol might, in the first place, be developed by best-available knowledge from international experts in the field,^37^ before examining the reliability and validity of the protocol.^38^

## Conclusion

To be able to obtain and maintain proficiency in performing the ABI examination, practical training with repetition and personalized feedback are required. However, the level of certainty for the evidence was judged to be very low. There is a need to develop an inter-societal examination protocol agreed upon between vascular bodies for vascular examination of the arterial circulation of the lower extremities.

## Supplemental Material

sj-docx-1-mde-10.1177_23821205241303560 - Supplemental material for Practice and Evaluation of Competence in Assessment of Arterial Circulation of the Lower Limbs among Medical Students and Physicians in Training – A Systematic Review Supplemental material, sj-docx-1-mde-10.1177_23821205241303560 for Practice and Evaluation of Competence in Assessment of Arterial Circulation of the Lower Limbs among Medical Students and Physicians in Training – A Systematic Review by Martin Macek, Frida Eek, Axel Wrede, Talha Butt and Stefan Acosta in Journal of Medical Education and Curricular Development
